# *Let all know*: insights from a digital storytelling facilitator training in Uganda

**DOI:** 10.1080/16549716.2021.1933786

**Published:** 2021-07-06

**Authors:** Tingting Yan, Michael Lang, Teddy Kyomuhangi, Barbara Naggayi, Jerome Kabakyenga, Wasswa William, Scholastic Ashaba, Clementia Murembe Neema, Manasseh Tumuhimbise, Robens Mutatina, Deborah Natumanya, Jennifer L. Brenner

**Affiliations:** a Department of Paediatrics and Community Health Sciences, Cumming School of Medicine, University of Calgary, Calgary, Canada; b Faculty of Nursing, University of Calgary, Calgary, Canada; c Common Language Digital Storytelling, Calgary, Canada; d Maternal Newborn and Child Health Institute, Mbarara University of Science and Technology, Mbarara, Uganda; e Faculty of Interdisciplinary Studies, Mbarara University of Science and Technology, Mbarara, Uganda; f Department of Biomedical Sciences and Engineering, Mbarara University of Science and Technology, Mbarara, Uganda; g Department of Psychiatry, Mbarara University of Science and Technology, Mbarara, Uganda; h Department of Human Development and Relational Sciences, Mbarara University of Science and Technology, Mbarara, Uganda; i Faculty of Business and Management Sciences, Mbarara University of Science and Technology, Mbarara, Uganda; j Department of Computer Science, Mbarara University of Science and Technology, Mbarara, Uganda

**Keywords:** Digital storytelling, DST, Uganda, health promotion, participatory action research, health communication

## Abstract

**Background:**

Digital storytelling (DST) is a participatory, arts-based methodology that facilitates the creation of short films called digital stories. Both the DST process and resulting digital stories can be used for education, research, advocacy, and therapeutic purposes in public health. DST is widely used in Europe and North America, and becoming increasingly common in Africa. In East Africa, there is currently limited in-country DST facilitation capacity, which restricts the scope of use. Through a Ugandan-Canadian partnership, six Ugandan faculty and staff from Mbarara University of Science and Technology participated in a pilot DST facilitation training workshop to enhance Ugandan DST capacity.

**Objective:**

This Participatory Action Research (PAR) study assessed the modification of DST methodology, and identified the future potential of DST in Uganda and other East African settings.

**Methods:**

In the two-week DST Facilitator Training, trainees created their own stories, learned DST technique and theory, facilitated DST with community health workers, and led a community screening. All trainees were invited to contribute to this study. Data was collected through daily reflection and journaling which informed a final, post-workshop focus group where participants and researchers collaboratively analyzed observations and generated themes.

**Results:**

In total, twelve stories were created, six by trainees and six by community health workers. Three key themes emerged from PAR analysis: DST was a culturally appropriate way to modernize oral storytelling traditions and had potential for broad use in Uganda; DST could be modified to address ethical and logistical challenges of working with vulnerable groups in-country; training in-country facilitators was perceived as advantageous in addressing community priorities.

**Conclusion:**

This pilot study suggests DST is a promising methodology that can potentially be used for many purposes in an East African setting. Building in-country DST facilitation capacity will accelerate opportunities for addressing community health priorities through amplifying local voices.

## Background

### Digital storytelling and public health

The digital age has transformed how stories are told and consumed across cultures [1]. Digital storytelling (DST) is a methodology that guides participants in the creation of short 2–5 minute movies using a voiceover, images, video, and music to tell the story of their experiences [2]. DST was pioneered in the early 1990’s by Dana Atchley and Joe Lambert at the Center for Digital Storytelling based in California (now known as the StoryCenter) [3], and has gained widespread attention in North America and Europe [4,5].

The community-based, participatory nature of DST allows it to ‘sit at the nexus of research, community engagement, and narrative’ [6, p.130] and therefore its potential uses are varied [6]. In public health, DST has been applied towards education [7], advocacy [8], research [1], and as a therapeutic tool [9], with many projects pursing multiple goals simultaneously [1]. One systematic review has suggested five general categories of outcomes where DST shows promise for public health initiatives: a) digital stories as ‘counter-narratives’, b) knowledge translation, c) preservation of cultural heritage, d) community development, and e) participatory research with marginalized groups [1].

While there are many possibilities for DST in public health, there are also ethical considerations that must be navigated [10]. Specific challenges include recruitment of storytellers and consent of parties represented in the stories, the potential of a facilitator shaping a participant’s story, confidentiality, and the potential of a story to harm the storyteller [10]. Thus, skilled, professional DST facilitators are essential as they bring awareness of ethical challenges and strong ethical practice to the storytelling process [10]. Indeed, all DST outcomes are closely tied to the quality of facilitation [2] as DST is fundamentally a co-creative process [11].

### Public heath digital storytelling in African contexts

DST has already been used in Africa with positive results in the fields of education and research [5,12–14], social justice [1,15–18], and public health [19–24]. Additionally, DST can help capture insights from marginalized groups, who are underrepresented in traditional research approaches [3]. African oral storytelling traditions are highly compatible with the structure of digital stories despite the differing lengths [16]. The 2–5 minute DST limit is primarily due to the time constraints of a DST workshop, but ultimately the short and succinct nature of digital stories allows for easier dissemination to a broad audience [2]. However, DST has developed traction more slowly in Africa than North America and Europe. Within Africa, there is more peer-reviewed documentation of DST initiatives in the single country of South Africa than what is available in all the countries of East Africa [5,12,14,20,23].

Specifically, in an African public health context, DST has been used to promote discussions around sexual health. Mnisi (2015) and Treffry-Goatley et al. (2016) used DST to understand how community members experienced and dealt with stigma from HIV/AIDS in South Africa [20,21]. Treffry-Goatley et al.’s digital stories were screened 151 times in a follow-up study and the authors found that DST successfully increased dialogue around HIV and antiretroviral therapy [20,23]. Africaaid, a non-governmental organization (NGO) in Harare, Zimbabwe, piloted a ten-day DST workshop with 12 HIV-positive adolescents [22]. Willis et al (2014) evaluated the impact of DST on the Africaaid workshop participants, and found that they were able to make positive reflections on difficult experiences, and regain a sense of control over their lives [22]. Additionally, six of the participants eventually released their stories to help train healthcare workers in Zimbabwe [22]. Hill (2008) took the StoryCenter’s Silence Speaks project to Uganda to conduct DST with rural women with obstetrical fistulas, to generate awareness around the issue. Hill (2008) recognized the need to adapt the North American developed Silence Speaks workshop to address the language, literacy, and technology challenges in the rural Ugandan context [23].

### DST facilitator training in East Africa

Building in-country capacity to facilitate DST is essential for its scale up as a valuable public health promotion tool in East Africa. A shift from cross-cultural DST facilitation with East African storytellers to increase in-country facilitator expertise could boost opportunities and quality. Indeed, Beran et al. (2017) describe how people in low and middle income countries are better placed to define issues of importance to their communities compared to people from high income countries, who may fund global health initiatives according to their own interests [25]. In-country leaders are also more likely to have buy-in from the community and policymakers, which can help develop more sustainable solutions [25]. Currently, documented East African DST initiatives are primarily led by North American-based organizations (e.g. StoryCenter) in partnership with in-country teams. Some country-based capacity initiatives for DST facilitation have been documented; in 2018, StoryCenter trained JSI Research and Training Institute staff in Nairobi, Kenya to help community youth share their HIV-related stories [26]. Also in 2018, UNICEF selected youth from a variety of African countries including Uganda, Kenya, Zambia, and Mozambique to attend a DST workshop [27,28]. These youth learned how to research and produce stories, and how to pitch these stories to commissioners [28]. However, in-country expertise remains relatively limited and there are gaps in documenting experiences and creating guidelines for facilitating DST in an East African setting.

In 2018, members of the Maternal, Newborn, and Child Health Institute (MNCHI) at Mbarara University of Science and Technology (MUST) were introduced to DST through a workshop hosted in partnership with the University of Calgary Cumming School of Medicine. The institutions subsequently partnered on an inaugural DST program with support from Wellcome Trust, UK, with the goal of building MUST’s capacity as a Ugandan-based DST centre, capable of facilitating culturally-relevant storytelling, training, research, and advocacy. This study used a Participatory Action Research approach with DST facilitation workshop participants, to understand how to modify the North American developed DST facilitator training model, and to identify the future potential of DST in Uganda and related East African contexts.

## Methods

### DST facilitator training

The DST Facilitator Training Workshop (Level 1; see Table A1) was developed and co-facilitated by one of the authors (ML, commonlanguagedst.org) incorporating eight years of DST facilitation experience and ongoing academic research [2,29,30]. A semi-structured four-step DST facilitation approach (Finding, Telling, Crafting, Showing) developed by Lang et al. (2019) guided curriculum development and overall structure of a 10-day, hands-on workshop held in Mbarara, Uganda, in July 2019.

The workshop engaged participants in preparing their own digital stories using *DaVinci Resolve Studio 16 s*oftware on MacBook Pro computers (days 1–2), followed by didactic lectures, group discussions, and participatory activities to explore the theoretical, logistical, and ethical considerations of DST facilitation with focus on public health and academic contexts (days 3–4). On day 5, stories were screened and discussed with the workshop group and selected staff from MNCHI. From day 6–10, participants worked directly with volunteer storytellers recruited from past MNCHI community-based health projects. Workshop participants worked one-to-one guiding volunteer storytellers through all four story creation phases. On day 10, a community DST screening was held with a small audience of invited academics from MUST and staff from the MNCHI.

### Participant recruitment, data collection, and analysis

All DST Facilitator Training workshop participants were invited to take part in this participatory action research (PAR) study. PAR involves collaboration between participants and researchers to generate knowledge through a cycle of collective study commitment, reflection, collective action, and shared analysis and dissemination of findings [31]. Workshop participant and DST trainer (ML & TY) reflections were gathered and analyzed using the following three processes:

- Daily reflective journals: At the end of each workshop day, each participant reflected on open-ended, pre-determined questions regarding their workshop experiences. Journaling prepared participants to engage fully in the evaluative discussion (see iii) below.
- Field notes: The DST trainers recorded field notes throughout the workshop related to specific emerging logistical, curriculum, and contextual considerations.
- Evaluative discussion: Following the workshop, participants shared perspectives during a 2 hour focus group guided by PAR evaluative discussion methodology [32]. Participants were presented with guiding questions, to generate discussion about (1) adaptation of the DST facilitator training curriculum for the Ugandan context, and (2) cultural relevance and potential opportunities for DST in surrounding communities. Participants initially formed pairs or trios to discuss observations and reflections from their journal entries. Thematic analysis was conducted when they rejoined as a larger group and identified common themes.

All of the data sources were brought together through the final evaluative discussion. The daily reflective journals and field notes were used to inform the discussion which was then used as the primary data analyzed for the study. After documenting the themes that were co-identified through the evaluative discussion, the themes were cross-checked with field notes from the DST trainers.

With regards to ethical considerations, PAR raises the concerns of confidentiality. This was mitigated through up-front discussion with the team about the level of comfort with a participatory approach to data collection and analysis, rather than using traditional qualitative research methods such as numeric identifiers to preserve confidentiality. Other common ethical principles that apply to PAR include informed consent, favourable risk-benefit ratio, and respect for participants, which were all met by this study [33]. Ethical clearance was obtained from both the University of Calgary and Mbarara University of Science and Technology.

## Results

A total of six individuals (two male, four female) participated in the DST Facilitator Training Workshop. The participant group was multidisciplinary, representing the Faculty of Interdisciplinary Studies (2), Applied Sciences and Technologies (1), Computing and Informatics (1), Business and Management Sciences (1), and Maternal, Newborn, and Child Health Institute (1). Workshop participants were university lecturers (5) or academic project field staff (1). Prior to the workshop, three participants described themselves as ‘not at all familiar’ with DST; three described their DST exposure as ‘slightly familiar’.

All six participants were involved in daily reflective journaling and the evaluative discussion. Each participant successfully created one digital story (DS) of their own topic choice and also successfully facilitated the creation of one DS with a volunteer storyteller. Resulting participant DS topics revolved around higher education (n = 4), personal health (n = 1) and gender equality (n = 1). The volunteer storyteller DS topics (n = 6) all focused on health-related experiences within the health system or in the community. For examples of volunteer stories, please see Kenneth’s story and Rose’s story.

Despite some technological challenges and a heavy content load, participants described the DST Facilitator Training Workshop as leading them to feel ‘comfortable’, ‘confident’, ‘empowered’, and ‘enlightened’. Participants appreciated review of the basic theoretical components of DST and the flexible structure of the four-step DST facilitation approach (finding, telling, crafting, and showing) which supported creation of stories for them and for the volunteer storytellers. Participants commented that teaching, group discussions, and DST ethics scenarios/activities were informative and well presented.

### Theme 1: there is great potential for DST in Uganda

Participants expressed that employing DST on a wider scale in the Ugandan context is ‘long overdue’, supported by the subthemes of cultural appropriateness, attractiveness of a strengths-based approach to knowledge sharing, and good fit with the digital revolution occurring in East Africa. Many examples of potential Ugandan DST applications were identified including fundraising for vulnerable groups, disseminating research results, advocating to decision makers, and promoting community health practices.

Participants described how DST has a good fit within the Ugandan oral storytelling culture. Through addition of music and imagery and with potential to extend a story’s reach beyond an in-person audience, DST could modernize the traditional storytelling approach. DST might also serve to enhance preservation of Ugandan culture as a means of passing knowledge between generations. Additionally, DST could challenge and add to didactic education methods like lectures and rote memorization which still dominate pedagogical approaches in Uganda.

Participants described DST’s ‘strengths-based orientation’ as an opportunity to highlight community successes as well as a tool to help achieve community and individual goals. They saw this in contrast to simply illustrating and cataloging challenges, which is common practice with other media formats. Participants anticipated that DST could increase bilateral knowledge exchange between Uganda and North America/Europe, inspiring even those from different income and cultural backgrounds to learn from Ugandan community-based public health promotion success.

Finally, the recent rise in internet use and smartphone accessibility provides a beneficial environment for increased East African DST use. One participant explained that DST could be shared through a new digital tool he was developing for video streaming in low bandwidth settings. Participants strongly believed that Ugandans from all backgrounds would be interested in creating and watching digital stories.

### Theme 2: reaching vulnerable groups using DST presents unique opportunities and challenges

Participants believed DST could enable illiterate and other vulnerable community members to better receive and share knowledge. For this to occur effectively, participants emphasized that DST must involve in-country DST facilitators working within their own cultural, language and operational context. DST facilitators need to speak the language of the storyteller, hold community trust, and navigate context-specific ethical considerations. Hence, training Ugandan DST facilitators was perceived as far more suitable than partnering with DST facilitators from outside the country to create digital stories in Uganda.

When working with vulnerable communities, conventional North American developed DST methodology may require adaptation to surmount logistical challenges such as limited technology access. Importantly, setting-specific ethical challenges are complex and must be carefully considered in advance. Workshop participants identified the needed modifications for this setting. Low-literacy storytellers should be provided with alternatives to written scripts for voiceovers; rural storytellers with limited smartphone access could be supported to collect suitable images/photos. Story creation format and timelines (Finding Phase) [2] could be extended to enable facilitators to accommodate travel time and relationship building with storytellers especially for vulnerable populations, where trust building, developing clear and shared objectives, and understanding of process and story sharing implications (including screenings and release) is critical yet may require more time. Extension of time for voiceover recording (Telling Phase) which involves capturing appropriate photos and completing the editing process (Crafting Phase) would especially benefit a largely rural or low-literacy population. Workshop participants estimated that a single digital story might take up to a week to complete when working with a storyteller from a rural or vulnerable group, compared to the two and a half days allotted in this DST Facilitation Workshop or three-day StoryCenter model [3].

### Theme 3: the way forward for DST and DST Facilitator trainings is in-country driven

Specific recommendations for increasing DST uptake in the Ugandan context were identified. Participants suggested a residential workshop format could promote a more immersive environment; development of a virtual platform/library to host locally created stories could increase opportunities for story sharing and screenings; ‘paired’ facilitators could work together to support preparation of digital stories–a ‘narrative storytelling lead’ and ‘information technology lead’ could work together in the absence of one individual with both narrative storytelling and information technology strengths.

Participants expressed that despite the general appropriateness of DST in an East African context, engaging other potential facilitators from outside the southwest Ugandan region would require additional consideration of cultural and linguistic differences that exist across this large and diverse area of the continent. Participants expected that some similar challenges such as technology limitations and low literacy. However, existing East African university and non-academic partnerships could be leveraged to broaden DST impact.

## Discussion

DST is a rapidly expanding methodology; this is the first documented facilitator training in East Africa that assessed both the modification of DST and the future potential in Uganda. A two-week, North American-developed DST training workshop did not require significant content adaptation for Ugandan academic participants and resulted in quality digital stories. However, the DST training workshop did require logistical adaptation for delivery of the content, that was addressed under Theme 2 (Results). DST was perceived as highly culturally appropriate, feasible, and usable for the Ugandan setting. Participants identified potential exciting DST opportunities within and beyond their personal academic work. Moving forward, Ugandan leadership of DST programming was seen as critical for DST expansion, relevance and ethical use in the Ugandan setting, especially with vulnerable populations. Workshop participants were well-positioned to have buy in from local community members to conduct DST, and better placed to define the issues of importance where digital stories could be used with high impact, compared to out-of-country leadership.

This study supports previously documented East African digital experiences in the not-for-profit sector for HIV/AIDS awareness and health promotion [22,23,34], women’s empowerment [35], and indigenous advocacy [16,17]. However, based on academic and grey literature, most prior documented DST production has occurred via North American-East African not-for-profit partnerships that focused on producing stories on specific themes related to organizational mandate, rather than training in-country facilitators [17,18,22,23,34,35]. To our knowledge, this is the first East African-based initiative to build in-country DST facilitation capacity within an academic setting. This study, and its focus on modifying and contextualizing a North American DST facilitation training model in partnership with in-country academics, opens even broader opportunities for East African DST for research sharing, public health promotion and community-driven advocacy.

Challenges encountered during this pilot DST Facilitator Training Workshop were similar to those previously identified by other DST researchers in Africa. Setting-relevant technological and infrastructure challenges were mitigated within course timelines, and ethical considerations were identified and managed. Similar to Dreyer et al. (2017) and Gogela & Ntwasa (2015) who described how student DST producers had challenges navigating video editing software [5,14], our DST course participants had variable experience with common video production software packages. Other researchers who have worked with low-literacy populations in Africa have described a similar need to adapt production timelines and processes; Bidwell et al. (2010) who created digital stories in South Africa, described challenges in voiceover writing [36]. Villager stories were prompted by images or landscapes, and they had a difficult time thinking of a story without these aids. In Uganda, Hill et al (2008) trained their interpreters to co-construct stories to support low-literacy creators [23]. Developing ‘made-in-Africa’ and low-literacy processes to meet emerging needs is critical for DST to thrive in East Africa.

All workshop participants worked in an academic environment. This is both a study opportunity and a limitation. Participants represent a highly motivated group with qualitative and narrative inquiry skills, expertise in technology, or both. Thus, uptake of concepts and content was likely quicker and possibly more effective in this group compared to those with less pre-workshop experience. Since participants had previous exposure to other forms of media to support advocacy for vulnerable groups, research dissemination, and public health promotion, they were well-positioned to contrast benefits and challenges of DST. In particular, participants described how DST applied more engaging storytelling techniques than static media (for example, photovoice), and how high-quality video productions were particularly prized in their context. They also described DST as most similar to traditional storytelling. Another strength was considerable participant experience with community-based work and knowledge sharing; experiences related to consent, health literacy, community entry and ethics were incorporated into DST practice and recommendations. Still, further perspectives from community members and storytellers, especially the most vulnerable themselves, will be critical moving forward to best understand key considerations, limitations and potential for DST. Finally, Ugandan facilitators shared language and culture of the participants, and were also fluent in English. Their background allowed for the stories to be translated between English and Runyankole using closed captioning and this was helpful for sharing the story in different settings.

This workshop was developed by (ML) and co-facilitated by (ML & TY) Canadian DST trainers. The most effective and suitable training and practice for DST in Uganda will require more hands-on experience and exposure by East African team members. Currently, development of local community stories, and team leadership in additional upcoming workshops, is underway. Documentation and ongoing evaluation of experiences will help inform future DST especially in creation of increasingly challenging stories with vulnerable storytellers and in communities and individuals where a pre-existing relationship is lacking.

This study is important because it affirms DST as a highly promising methodology to share critical and untold stories from and within Uganda when fully supported by an in-country team. Investing in the development of Ugandan DST facilitation capacity will likely be more effective than partnerships involving out-of-country leadership to move DST to its full potential and can better support DST scale-up within East Africa. Development partners, research institutions, advocacy groups and health promoters should pay attention to this emerging methodology, since Ugandan DST facilitators trained in this study and other public health researchers [1,6] believe it has widespread potential for powerful change.

## Conclusion

This PAR study assessed considerations for modifying the North American developed DST facilitation model to Uganda, and identifying the future potential for DST in Uganda and related East African contexts. The results indicated great potential for Ugandan DST use. To maximize success, a strong in-country DST facilitation team is critical. Since the initial workshop described in this manuscript, newly trained DST facilitators have supported creation of new digital stories, practiced key skills, and identified new issues. A follow up training to manage emerging technical, practical and ethical issues was conducted and ongoing mentorship is being provided. Future manuscripts will report on the results of this ongoing collaboration as well as the various public health topics, uses, and impacts of the stories that are being created. The new DST facilitation team, based at MNCHI in Mbarara continues to build skills, showcase stories and are developing an online digital story library. Documentation on emerging advanced level training, ethical challenges, low literacy strategies and technological solutions will hasten DST effectiveness and scale up for Uganda. We hope DST can provide an important opportunity for the most vulnerable in East Africa to speak for themselves.

## Paper context

Digital Storytelling (DST) has potential for greater impact as a health promotion, research and advocacy tool in East Africa. In Uganda, DST is primarily conducted by out-of-country facilitators. This study finds that building in-country DST facilitation expertise can enhance the application of DST to community priorities, and help overcome logistical challenges including technology and literacy considerations. Further documentation of DST best practices in different cultures and settings, especially amongst vulnerable groups, will strengthen DST globally.

## Appendix A: Workshop Outline

**Table A1.** Outline of 10 day DST facilitator training at Mbarara University – July 2019

Day	Session	Time	Topics/Activities
0	Pre-Workshop Webinar	3 hrs – Webinar	Overview of workshop, objectives, expectations & DST Facilitator Training research project, DST Research and Practice, Finding the Story and Basic Story Structure, specific screenwriting tips and tools to structure a story for greatest impact.
1	Digital Storytelling Workshop – Day 1	8 hrs – In Person	Story Circle, Tutorial #1 – Video Editing Basics & Image Selection, Finalizing Stories & Voiceover Recording
2	Digital Storytelling Workshop – Day 2	8 hrs – In Person	Tutorial #2 – Advanced Video Editing Tools, Digital Story Editing & Music Selection, Finalizing Stories, Digital Story Screening
3	Digital Storytelling Facilitator Training Day 1	8 hrs – In Person	Debrief of DST Workshop Experiences, Overview of History of DST, Theoretical Foundations of DST, Foundational Ethical Principles of DST, DST Project Planning, DST Facilitation Theory #1 – Finding & Telling
4	Digital Storytelling Facilitator Training Day 2	8 hrs – In Person	Training Review, DST Facilitation Theory #2 – Crafting, DST Facilitation Theory #3 – Sharing, DST in a Ugandan Context
5	Digital Storytelling Facilitator Training Day 3	5 hrs – In Person	Review of entire DST Training Week, Final Digital Story Edits incorporating additional learning, Digital Story Screening & Discussion with entire DST project team.
6–7	Digital Storytelling Facilitator Practice Day 1	8 hrs – In Person 3 volunteers/day	DST workshop using same outline as above facilitated by participants for volunteer storytellers from the community with the support of Trainer and Assistant.
8–9	Digital Storytelling Facilitator Practice Day 2	8 hrs – In Person 3 volunteers/day	DST workshop using same outline as above facilitated by participants for volunteer storytellers from the community with the support of Trainer and Assistant.
10	Focus Group and DST Film Screening & Presentation	8 hrs – In Person	DST Film Screening & Discussion at Mbarara University, Final Discussion with Project Team, DST Facilitator Training Research Project Focus Group, Next Steps, Closing.

## Appendix B: Participatory Action Research Questions

Pre-Workshop Questionnaire

1. What are your goals and expectations from the DST workshop?
2. Why did you decide to be part of this workshop?
3. What concerns do you have about the DST workshop and the process of DST?
4. How familiar are you with DST? (please check one box)
  **Table:**
  Not at all familiar	Slightly familiar	Moderately familiar	Veryfamiliar	Extremely familiar

Daily Reflective Journals

*Days 1–5*

Please read the following questions and provide feedback to the best of your abilities.

1. To what extent do you think the learning objectives stated at the beginning of the session were met? (Please check one box)
  **Table:**
  Strongly disagree	Disagree	Neutral	Agree	Strongly agree
2. Do you feel today’s content was valuable for you?
3. What were the greatest strengths of the workshop today?
4. What were the greatest challenges in the workshop today, and what are your suggestions on how to improve upon them?
5. What was your main learning or take-away from the session?
6. Are there any other reflections you’d like to add, or questions to ask during the focus group?

*Days 6–10*

Please read the following questions and provide feedback to the best of your abilities.

1. Did you feel sufficient support throughout today’s working session?
2. Do you feel today’s content was valuable for you?
3. What were the greatest strengths of the workshop today?
4. What were the greatest challenges in the workshop today, and what are your suggestions on how to improve upon them?
5. What, if anything, surprised you about creating a DST (compared to learning about how to create it)?
6. Is there anything you wish you had learned in Week 1 of the workshop that would have better prepared you today?

PAR Focus Group Questions

Topic 1: How effective was the DST facilitator training workshop?

1. How familiar do you feel with the DST methodology after the workshop?
2. What did you expect the DST facilitator training to be like? Was it below, at, or above your expectations?
3. What were the biggest strengths of the DST workshop this week?
4. What were the biggest challenges in the DST workshop this week, and how do we overcome these challenges for future workshop participants?

Topic 2: How culturally appropriate/sensitive is the DST methodology, and the DST facilitator training?

1. How do you think DST differs from other methods of education, advocacy, research, and therapy that have been used in your community in the past?
2. Beyond the methods listed above, how else do you think DST could be used in Uganda?
3. How does DST fit in with the cultural traditions of the Ugandan community?
4. Who among Ugandans would be interested in using or viewing DST?

Topic 3: How feasible is it to use DST in your own work, and in Uganda, and in Africa?

1. What are your current perceptions of DST initiatives in Africa?
2. How prepared would you feel to conduct DST with a member of your community? How about training others on using DST?
3. What benefits do you foresee in using DST in the Ugandan/East African settings?
4. What challenges do you foresee in using DST in the Ugandan/East African settings?

Wrap-up

1. Out of everything we talked about in each topic, what single factor is most important to you?
2. Is there anything you think we have missed during this discussion?
3. What would you want to take away, keep, or add to the workshop?
4. What will you tell others about DST?

## Appendix C: Brief Biographies of the Facilitators-in-Training

Barbara Naggayi, MSc is a PhD Candidate and lecturer in the Faculty of Interdisciplinary Studies, Mbarara University of Science and Technology, Uganda.

Robens Mutatina, BSc is a Field Officer with Healthy Child Uganda, Maternal, Newborn, and Child Health Institute, Mbarara University of Science and Technology, Uganda.

Dr. Manasseh Tumuhimbise, PhD, is a Lecturer in the Faculty of Business and Management Sciences, Mbarara University of Science and Technology, Uganda.

Dr. Neema Murembe, PhD, is the head of the Department of Human Development and Relational Sciences in the Faculty of Interdisciplinary Studies, Mbarara University of Science and Technology, Uganda.

Deborah Natumanya, MSc, is a PhD Candidate and lecturer in the Department of Computer Science, Mbarara University of Science and Technology, Uganda.

Wasswa William, MMedSc, is a PhD Candidate and Assistant Lecturer in the Department of Biomedical Sciences and Engineering, Mbarara University of Science and Technology, Uganda.
